# On the Ordeal Bean of Calabar; Its Action on the Human Body, Compared with That of Woorara and Conia

**Published:** 1864-08

**Authors:** 


### *On the Ordeal Bean of Calabar; its Action on the Human Body, compared with that of Woorara and Conia.*

The Ordeal Bean of Calabar is a vegetation of the Gold Coast. The natives call the plant *Eséré*, and it was by the missionaries named the ordeal bean, in consequence of its being given to persons suspected of witchcraft, with a view of detecting their innocence or guilt. Both in taste and appearance it resembles the common white haricot bean.

In a paper read before the Royal College of Physicians and Surgeons by Dr. Harley, the author draws the following conclusions: 1. The ordeal bean may cause contraction of the pupil when taken internally, as well as when applied locally, even. 2. That atropine and the Calabar bean are physiologically antagonistic. 3. That the ordeal bean paralyzes the motor nerves, and leaves the intelligence and muscular irritability unimpaired. 4. That it excites the salivary and lachrymal secretions. 5. That it destroys life by paralyzing the nerves supplying the respiratory muscles—being, in fact, a respiratory poison. 6. Although it may weaken the heart, it neither stops the circulation nor arrests the heart's action. It is not, in fact, a cardiac poison. 7. It is closely allied in its effects to woorara and conia, most closely, perhaps, to the latter; but it differs from both in its tendency to produce muscular twitchings, and in its power of producing contraction of the pupil. Neither woorara or conia exert, generally or locally, any such effect on the iris. 8. The ordeal bean will prove a most valuable addition to the pharmacopoeia, by not only giving us a useful myopic, but also a powerful anodyne, capable of soothing nerve irritation without either destroying intelligence or endangering life by arresting the heart's action.

Mr. Solberg Wells was somewhat surprised that, in enumerating the peculiar properties of the Calabar bean, Dr. Harley had not called more attention to its irregular power of causing contraction of the ciliary muscle, and thus affecting the accommodation of the eye, as this was of far greater importance than its action upon the pupil. The impairment of vision which follows the application of atropine is not due to the dilatation of the pupil, but to the paralysis of the accommodation. This is proved by the fact, that if we employ a sufficiently week solution of atropine, so that the constrictor pupillæ alone, and not the ciliary muscle also, is paralyzed, vision will be but very slightly impaired. Now, the Calabar bean possesses the peculiar power of not only causing contraction of the pupil, but also of the ciliary muscle, thus changing the normal into a short-sighted eye. It also counteracts the paralyzing effects of atropine upon these muscular structures. With respect to the local action of the bean he might remark, that Professor Czermak and he had been trying its effects upon the eyes of rabbits, directly after decapitation, and they had found that it produced marked contraction of the pupil within about twenty minutes of its application.

Mr. Hulke communicated briefly the results of three experiments which had been made with the alcoholic extract of the bean on patients in the Royal London Ophthalmic Hospital. The first patient had paralysis of both third (cranial) nerves, and mydriasis from syphilitic periorbitis. Two hours after the application of the extract to the right eye, the nearest point of distinct vision was sixteen and a half inches, and the diameter of the pupil was one line, the proximate point having been previously twenty-six inches, and the pupil two and a half lines broad. In the same time the near point of the left eye had become twelve and a half instead of twenty inches, and the pupil one line instead of three lines across. The second patient had paralysis of the left-third crania nerves, with mydriasis, of four years' standing, the consequence of traumatic periorbitis with abscess. In one hour the proximate point had become six instead of eight and a half inches, and the pupil had contracted from three to three-quarters of a line. In the other unaffected eye the application of the extract effected, in the same time, an alteration of the proximate point from eight and a half to four inches, and reduced the pupil from one and a half to three-quarters of a line. The third was a case of paralysis of the left-third cranial nerve, with mydriasis from periorbitis, possibly rheumatic, which had been twice previously cured with iodide of potassium. In an hour the proximate point of distinct vision was brought from ten to five inches, and the pupil changed from three to three quarters of a line in diameter.

According to Mr. Ernest Hart, glycerine is the only proper solvent for the alcoholic extract of the bean, the watery solution undergoing rapid decomposition. The solution in which one minim corresponds to three grains of the bean answers best. It counteracts the effects of a solution of atropia, of three grains to the ounce, in dilating the pupil—inducing recontraction.

